# Editorial: The future of physiology: 2020 and beyond, Volume III

**DOI:** 10.3389/fphys.2023.1159406

**Published:** 2023-03-01

**Authors:** John D. Imig

**Affiliations:** Department of Pharmaceutical Sciences, University of Arkansas for Medical Sciences, Little Rock, AR, United States

**Keywords:** physiology, ion channels, biophysics, cell membrane, metabolic, obesity

*Frontiers in Physiology* launched in 2010 to provide a forum for the free exchange of ideas and to develop an international community of scientists working together to meet the challenge of integrating function from molecules to man. Several sections were initiated with the editors writing articles addressing the most pressing challenges and unanswered questions for their field of investigation. These articles set the direction and stage for the first decade of the journal. *Frontiers in Physiology* has allowed the worldwide community of scientists to exchange discoveries, ideas, and solutions that have ultimately led to improved health and quality of life. The continuing mission for *Frontiers in Physiology* will be to unite various physiology sub-disciplines to understand how the various components of an organism work together to maintain a healthy state and how disruptions in function lead to diseases. The physiology field is evolving fast. *Frontiers in Physiology* introduced Research Topics to address the challenges expressed by the editors. *Frontiers in Physiology* has launched over 1,800 Research Topics led by experts of the respective fields to capture this evolving and cutting-edge research. Now that it has been over a decade since Frontiers in Physiology was launched, it is time to reflect on what has been accomplished so far and what questions and Research Topic remain to be addressed. *Frontiers in Physiology* has expanded the number of sections and new section editors have taken the helms of those sections. Therefore, the goal of identifying Research Topics that permit collaboration of researchers across the world to accelerate the science in these physiological areas. In particular, the major Research Topic, unmet challenges, emerging technologies, and promising areas of research in each sub-discipline will be explored to inspire and to inform readers and researchers in the field of physiology for the year 2023 and beyond.

This Research Topic contains four articles that capture several directions that *Frontiers in Physiology* will be exploring. *Frontiers in Membrane Physiology and Biophysics* celebrated publishing 600 articles last year. This section has focused on the architecture, function, and cellular roles of biological membranes. Dr. Fahlke emphasizes the importance of membrane physiology to a variety of human diseases caused by dysfunctional membrane transport processes (Fahlke). An ever-increasing area of membrane physiology and biophysics is the identification of channelopathies that lead to human disease. There is a need to further explore membrane signaling in biological systems. Research areas to explore include the contribution of microvesicles to human health and diseases, computational and experimental approaches to investigate ion channels and transporters, and new techniques to determine membrane protein structures. A related article explores the function of ion channels and membrane potential in red blood cells (von Lindern et al.). Ion channels in erythrocytes as important contributors to physiology and pathophysiology is explored. This article proposes an approach that utilizes, molecular biology, *in vitro* erythropoiesis, state-of-the-art electrophysiological techniques, and channelopathies found in patient samples to evaluate erythrocyte ion channel function in health and disease. Major challenges in metabolic physiology were the focus of a third article (Imig). The importance of understanding metabolic physiology is highlighted by the alarming rates by which obesity, type 2 diabetes, metabolic syndrome, and liver diseases are increasing. Major areas that require further exploration are metabolism and metabolites, organ regulation in metabolic physiology, the epidemic of metabolic diseases, and metabolic adaptation to the environment. The last article highlights research that addresses grand challenges in metabolic physiology. The ability of minocycline to act in the hypothalamus to improve metabolic function in obese mice is demonstrated (Coker et al.). The findings of this experimental study found that minocycline decreased high fat diet-induced weight gain and improved insulin signaling by reducing inflammatory processes in the paraventricular nucleus, a key hypothalamic region regulating metabolic function. Taken together, these four articles highlight emerging areas in membrane physiology and biophysics and metabolic physiology.

*Frontiers in Physiology* has started a second decade of providing the exchange discoveries, ideas, and solutions that have ultimately led to improved health and quality of life. The field of physiology is ever evolving as new experimental and computational techniques allow for a deeper understanding of the genome, transcriptome, proteonome, metabolome, microbiome, and lipidome. The journal and sections will continue to seek articles that incorporate innovative techniques and approaches to meet the grand challenges in physiology. *Frontiers in Physiology* will undoubtedly attract the best science in the next decade that addresses major challenges in physiology.

